# Analysis of Risk Factors for Colorectal Cancer Associated with Ulcerative Colitis Using Machine Learning: A Retrospective Longitudinal Study Using a National Database in Japan

**DOI:** 10.3390/cancers17233752

**Published:** 2025-11-24

**Authors:** Miwa Hirai, Yasuhiro Kanatani, Takashi Ueda, Masaya Sano, Hiroaki Arai, Yurin Miyake, Naoko Tomita, Shota Nemoto, Hidekazu Suzuki

**Affiliations:** 1 Division of Gastroenterology and Hepatology, Department of Internal Medicine, Tokai University School of Medicine, 143 Shimokasuya, Isehara 259-1193, Japan; mhirai.tokai@gmail.com (M.H.); takashi_6_132@yahoo.co.jp (T.U.); m-sano@tokai.ac.jp (M.S.);; 2 Department of Clinical Pharmacology, Tokai University School of Medicine, 143 Shimokasuya, Isehara 259-1193, Japan; kanatani.yasuhiro.f@tokai.ac.jp (Y.K.);; 3 Industrial & Digital Business Unit, Hitachi, Ltd., Tokyo 101-0021, Japan; shota.nemoto.nt@hitachi.com

**Keywords:** ulcerative colitis, colorectal cancer, 5-ASA, artificial intelligence

## Abstract

**Background/Objectives:** Ulcerative colitis (UC) is a chronic inflammatory bowel disease that significantly increases the colorectal cancer (CRC) risk. This study used nationwide data on intractable diseases to clarify the clinical epidemiology of UC-related CRC in Japan. **Methods**: Patients diagnosed with UC between FY 2003 and 2011 were included. The relative incidence ratio (RR) was calculated using the standardized incidence ratio from the National Cancer Registry. To compare prognostic factors, outcomes were evaluated using the Cox proportional hazards model analysis for cancer occurrence, and a prognostic prediction model was developed using machine learning. **Results**: Among 78,556 patients with UC, CRC was identified in 141 patients. The RR of CRC peaked in both males and females in the 25–39 age group. Univariate analysis revealed several risk factors, including pseudo-polyps observed during endoscopy (hazard ratio 2.92, *p* = 0.001), abnormal crypt architecture (hazard ratio 3.14, *p* < 0.001), and dysplasia (hazard ratio 11.31, *p* < 0.001) in biopsy. Conversely, 5-ASA was associated with reduced CRC risk (hazard ratio 0.36, *p* = 0.003). The machine learning model categorized patients into three groups, demonstrating that the group with the highest number of patients with pancolitis had a significantly higher risk of CRC than did the other groups. **Conclusions**: Pseudo-polyps and dysplasia represent CRC risk factors in patients with UC. Additionally, machine learning analysis indicates that pancolitis in individuals in their 50s increases the risk of colon cancer, while proctitis in those in their 30s raises rectal cancer risk. These findings aim to enhance early detection and improve prevention efforts for UC-related CRC.

## Simple Summary

Ulcerative colitis (UC) significantly increases the colorectal cancer (CRC) risk. Nonetheless, only a few nationwide, long-term epidemiological studies have evaluated UC prognosis. Our study, which involved national surveillance of patients with UC, demonstrated that CRC risk in these patients was over 10 times that in the general population. This study identified several risk factors for CRC, including pseudo-polyps on endoscopy, dysplasia, and abnormal crypt architecture on biopsied specimens. Conversely, 5-aminosalicylic acid (5-ASA) reduced CRC risk in UC.

## 1. Introduction

Ulcerative colitis (UC) is a chronic inflammatory bowel disease that significantly increases colorectal cancer (CRC) risk [1]. Long-term chronic inflammation raises cancer risk by leading to genetic mutations and cellular stress, driven by enhanced reactive oxygen and nitrogen species and the induction of cytokines such as interleukin-6 and tumor necrosis factor-α [2]. CRC is a serious complication in patients with UC, as it is associated with high mortality rates and warrants crucial preventive management [3]. Despite the growing number of patients with UC worldwide, comprehensive, large-scale, long-term epidemiological studies on the incidence, timing of onset, and risk factors for CRC in these patients are lacking. Clarifying the clinical epidemiology of UC-associated CRC may lead to tailored CRC prevention strategies based on the pathophysiology of patients with UC.

Notably, CRC associated with UC exhibits specific clinical characteristics, including a preference for the distal colon, presence of pancolitis as a background condition, an endoscopic Mayo subscore of ≥2, and a non-polypoid appearance [4]. Additionally, CRC associated with UC tends to have a poorer prognosis compared to general CRC [5]. A multicenter cross-sectional study of patients with UC in Latin American countries who had been diagnosed for 8 years identified disease duration and inflammatory polyps as a CRC risk factor [6]. Importantly, the presence of inflammatory polyps is associated with dysplasia detection [7]. Furthermore, low-grade dysplasia occurring after age > 55 years is associated with an increased cumulative risk of colorectal cancer [8].

Dysplasia is a known cancer precursor; thus, establishing an effective surveillance system is vital for CRC early detection and treatment [9]. Chronic intestinal mucosal inflammation associated with UC may increase inflammatory cytokine release, potentially contributing to increased CRC risk in these patients [10].

Conversely, 5-ASA, a medication that maintains UC remission, has demonstrated efficacy in inhibiting mucosal inflammation and suppressing the expression of cyclooxygenase-2, epidermal growth factor receptor, and phosphorylated enzyme 2A, all of which are linked to CRC proliferation [11]. Therefore, 5-ASA may exert chemopreventive effects.

We conducted a retrospective longitudinal study involving patients with UC diagnosed between 2003 and 2011, focusing on those who developed CRC during the observation period, which lasted 2–10 years after registration.

This study aimed to clarify the relationship between clinical findings at registration, subsequent treatment decisions, and CRC occurrence. We used machine learning to develop a prognostic model that identified key factors associated with CRC.

## 2. Materials and Methods

### 2.1. The National Registry for Intractable Disease

Patients diagnosed with UC were eligible for a specified disease treatment program initiated in Japan in 1972 as a national registry to investigate the etiology and pathology of intractable diseases and to reduce patients’ medical expense burden. When applying for medical expense subsidies, patients submitted a consent form for data registration and use, together with an application form including their medical information. Certified patients were required to reapply and update their information annually. Consent for minors was provided by a guardian. After approval by a review committee, including gastroenterologists and the respective prefectural governors, personal information was anonymized and registered in the Ministry of Health, Labour, and Welfare (MHLW) database [12].

### 2.2. Description for Collected Data

The following demographic and clinical factors were obtained from application forms submitted by patients with UC: sex; date of birth; age at onset; date of first clinical visit; family history; clinical, radiological, endoscopic, and histologic findings; activities of daily living function; and treatment (Supplementary Table S1). Only patients who completed ≥2 years of follow-up after initial registration were included in the CRC prediction analysis (observation end date: 31 March 2014). Patients included in the analysis fulfilled the diagnostic criteria for UC established by the study group for intractable inflammatory bowel disorder, which align with those of the MHLW (Supplementary Text S1).

### 2.3. Definition of CRC

Patients with CRC were identified based on entries for “colon cancer” and “rectal cancer” in the “complications” section and free-entry section of the individual survey form; therefore, the CRC count was defined as the sum of colon and rectal cancer cases. Standardized incidence rates for colorectal and rectal cancers in the general population were obtained from the Cancer Statistics, Cancer Information Service, National Cancer Center Japan (National Cancer Registry, MHLW). To determine the relative incidence ratio (RR), we first calculated the expected value by multiplying the standardized cancer incidence rate for the general population by the number of patients with UC in each 5-year age group, and then divided the actual number of patients with CRC by this expected value.

### 2.4. Disease Distribution and Clinical Assessment

Disease distribution was determined using the Montreal classification as follows: E1, ulcerative proctitis; E2, left-sided UC; and E3, pancolitis-type UC. A case not in the Montreal classification system was defined as “Other” [13].

### 2.5. Machine Learning Model

A pointwise linear (PWL) model (B3 analytics, Hitachi Ltd., Japan) was implemented using PyTorch 1.5.1 (Linux Foundation) and Python 3.7.4 (Python Software Foundation) to predict remission induction after 3 years and enable patient stratification [14]. The PWL model is an explainable machine learning method that provides a weight vector tailored to each sample. The weights in a PWL model are calculated as nonlinear functions of the features via a neural network, unlike logistic regression.

The feature variables in the dataset were classified as binary, categorical, or quantitative (Supplementary Table S2). Binary variables were encoded as 1 or −1, while quantitative variables were normalized (mean = 0; standard deviation = 1). After encoding and normalization, missing values were imputed with zeros because zero-valued inputs do not change the output in the model’s weighted-sum layers, leaving the model’s interference unaffected.

To optimize hyperparameters and evaluate the model’s predictive performance, 10-fold double cross-validation (DCV) was performed. The model with the best prediction accuracy, measured by the highest area under the curve (AUC) on the test set in the 10-fold DCV, was used for patient stratification. Details of the optimal hyperparameters are listed in Table 1.

### 2.6. K-Means Clustering Algorithm for UC

**Figure 1:**
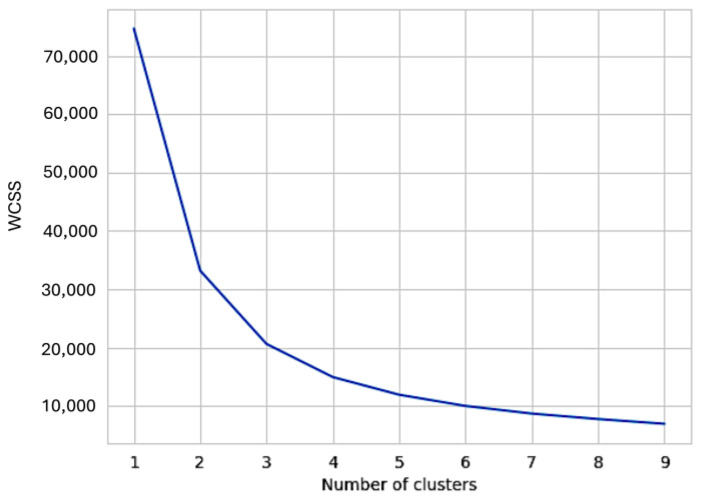
Optimizing the number of clusters using the elbow method. The relationship between the within-cluster sum of squares (WCSS) and the number of clusters obtained using the K-means method is displayed. The slope of the graph changes significantly around k = 3, indicating that the optimal number of clusters is 3.

Given UC heterogeneity, patients may have been divided into subgroups depending on their features. Therefore, clustering was performed using a weight vector tailored for each patient in the PWL model. The weights were stratified into *K* = 3 clusters using the *K*-means + algorithm (implemented in Scikit-learn version 0.24.2). The elbow method was used to select *K* (Figure 1) [15].

### 2.7. Statistical Analysis

Descriptive statistics are reported as counts (percentages) to describe patient characteristics. The Kruskal–Wallis one-way analysis of variance by rank test was used to compare cluster variables. All *p*-values are reported to three decimal places (e.g., *p* < 0.001). χ2 tests were used to compare categorical variables. Residual analysis was performed to determine which cell numbers in the cross-table represented sources of bias (*p* < 0.05) when significant bias was observed in a χ2 test (*p* < 0.05). Hazard ratios (HRs), 95% confidence intervals (CIs), and *p*-values were calculated using the Wald test. Cox proportional hazards analysis of prognostic factors was performed for each age at registration by selecting variables with *p* < 0.05 in univariate analysis. All analyses were performed using STATA version 18.0 (Stata Corporation LLC, College Station, TX, USA).

## 3. Results

### 3.1. Study Population

This study included 78,556 patients diagnosed with UC from the MHLW database between 2003 and 2011 (before the widespread use of biologics in Japan). The observation period ranged from a minimum of 2 years to a maximum of 10 years from the time of registration. For patients with confirmed cancer, data collection continued through the year of diagnosis. Table 2 displays the number of patients with UC who completed follow-up within 1 to 10 years after registration, categorized by fiscal year from 2003 to 2011. The rate of follow-up completion 1 year after registration was 47.9%, gradually increasing over the years and reaching 78.9% at the 10-year mark.

### 3.2. Standardized Incidence of Colon Cancer and Rectal Cancer in UC

**Figure 2:**
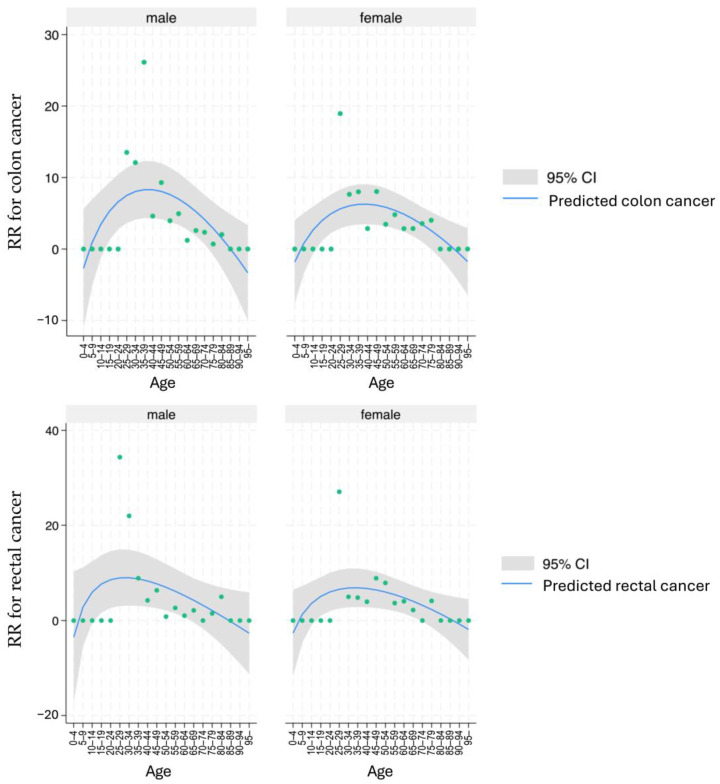
RR for colon or rectal cancer by age group. RR for colon cancer (upper) and rectal cancer (lower) in the general population with UC. RR, relative incidence ratio; CI, confidence interval.

Figure 2 displays the RR of cancer incidence among patients with UC, stratified by sex, cancer type, and age group, compared with the general population. For colon cancer, the RR was highest in the 25–39 age group for both sexes. Similarly, the peak for rectal cancer incidence occurred in the same age group for both sexes. After age 50, the RR for both cancer types declined. When comparing patients with UC to the general population, the RR for colon cancer was 3.64 and 3.99 for males and females, respectively. For rectal cancer, the RR was 3.96 and 4.75 for males and females, respectively.

### 3.3. Clinical Characteristics

Patients with CRC were older (54.5 vs. 41.3 years, *p* < 0.001) and had a later disease onset (50.7 vs. 38.5 years, *p* < 0.001) compared to those without; additionally, they had lower body mass index, red blood cell counts, hemoglobin levels, and albumin levels, alongside higher erythrocyte sedimentation rate (ESR). Endoscopic findings in the CRC group revealed decreased mucosal friability (85.2% vs. 92.7%, *p* = 0.001) and erosion (73.8% vs. 84.1%, *p* = 0.001), but an increased prevalence of pseudo-polyps (23.3% vs. 9.4%, *p* < 0.001). Moreover, histological examinations revealed that dysplasia (46.6% vs. 6.3%, *p* < 0.001) and abnormal crypt architecture (58.8% vs. 37.2%, *p* < 0.001) were significantly more prevalent in the CRC group than in the group without CRC. Regarding treatment-related factors, CRC incidence was significantly decreased in patients who used 5-ASA (73.2% vs. 93.8%, *p* < 0.001), while no significant differences were observed in those who used corticosteroids, immunosuppressive agents, or G-/L-CAP (Table 3).

### 3.4. Identifying CRC Risk Factors

Univariate and multivariate analyses were performed using Cox proportional hazards analysis of CRC outcomes (Table 4). Univariate analysis identified age at registration (HR = 1.05, 95%CI 1.03–1.06, *p* < 0.001), pseudo-polyps (HR = 2.92, 95%CI 1.58–5.43, *p* = 0.001) on endoscopic findings, abnormal crypt architecture (HR = 3.14, 95%CI 1.74–5.85, p < 0.001) and dysplasia (HR = 11.31, 95%CI 6.50–19.69, *p* < 0.001) on biopsy findings; and 5-ASA administration (HR = 0.36, 95%CI 0.18–0.71, *p* = 0.003) as risk factors. Multivariate analysis identified age at registration (HR = 1.09, 95%CI 1.03–1.09, *p* < 0.001) and dysplasia (HR = 8.53, 95%CI 4.30–16.9, *p* < 0.001) on biopsy findings as risk factors.

**Figure 3:**
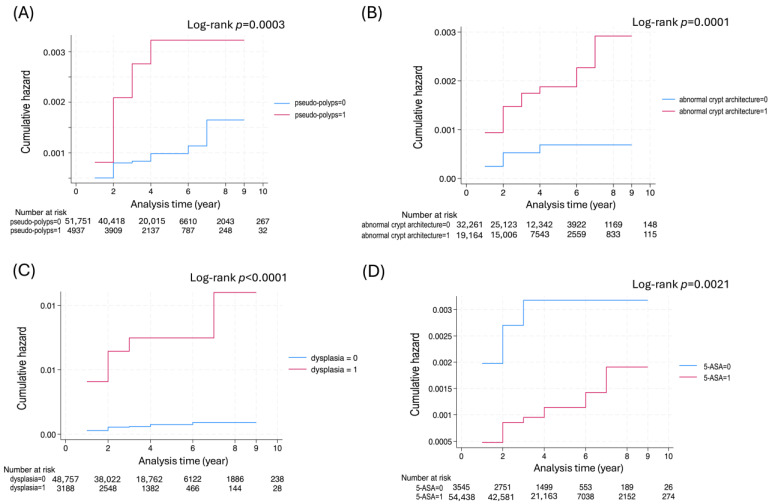
Nelson–Aalen estimates of the cumulative hazards for CRC. (**A**) pseudo-polyps, (**B**) abnormal crypt architecture, (**C**) dysplasia, (**D**) administration of 5-ASA: cumulative hazard for CRC according to presence (=1, red line) or absence (=0, blue line). 5-ASA, 5-Aminosalicylic acid.

Nelson–Alen estimates of cumulative hazards for CRC are displayed in the presence or absence of pseudo-polyps, dysplasia, and abnormal crypt architecture (Figure 3). The log-rank test demonstrated that patients with these findings exhibited a significantly increased CRC incidence. Conversely, 5-ASA use was significantly associated with a reduced risk of CRC (*p* = 0.0021).

### 3.5. Prediction Model

Machine learning predicted CRC outcomes and constructed a predictive model. On the test dataset, the model yielded an AUC of 1.00, a recall of 0.87 ± 0.12, a precision of 1.00, and an F-score of 0.93 ± 0.07. The model classified patients into three clusters: clusters 1, 2, and 3 included colon cancer, neither colon cancer nor rectal cancer, and rectal cancer, respectively. Demographic analysis demonstrated the youngest and oldest ages at registration in clusters 3 and 1: 33.9 ± 13.2 years and 53.7 ± 17.0 years, respectively. Regarding inflammation distribution, cluster 1 was a pancolitis type from the rectum to the cecum, whereas cluster 3 tended to concentrate in the rectum. The Mayo score at registration was highest in cluster 3 (4.8 ± 2.0) and lowest in cluster 1. Endoscopic findings exhibited a significantly lower incidence of pseudo-polyps in cluster 2. Histopathology showed the highest dysplasia frequency (9.1%) in cluster 3. Regarding treatment, cluster 3 had the lowest 5-ASA usage (85%), highest corticosteroid usage (41%), and highest immunosuppressant usage (2.3%) (Table 5).

**Figure 4:**
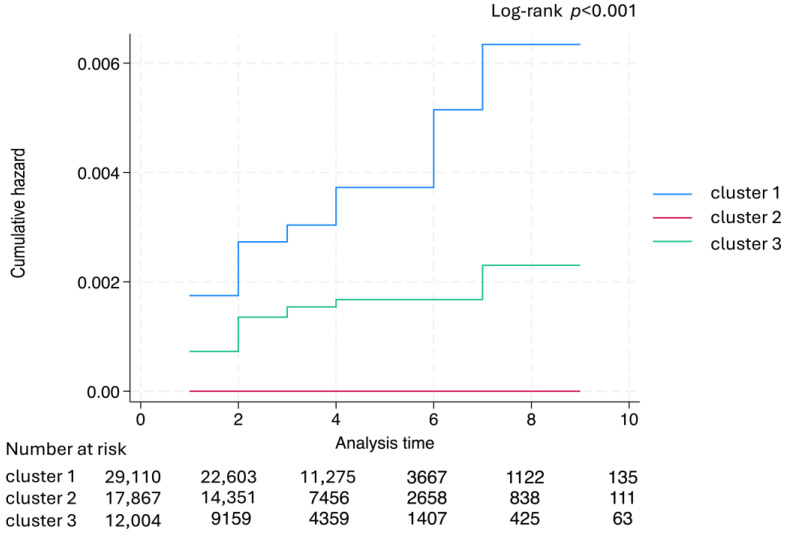
Nelson–Aalen estimates of the cumulative hazards for CRC for each cluster.

The Nelson–Aalen estimates of the cumulative hazards for CRC for each cluster are demonstrated in Figure 4. Among the three clusters, no CRC cases were observed in cluster 2, whereas cluster 1 showed a significant CRC risk increase over time compared with the other clusters (log-rank test, *p* < 0.001).

## 4. Discussion

Inflammatory bowel disease (IBD) is associated with increased CRC risk, with studies demonstrating that IBD-related CRC is typically diagnosed 15–20 years earlier than sporadic CRC [16]. Here, CRC incidence peaked in the 30 s for both sexes (Figure 2). Furthermore, a study using national Swedish health and census registers to compare CRC standardized incidence ratios between patients with IBD and the general population reported relative risks of 2.6 and 1.9 for male patients with IBD and their female counterparts, respectively, indicating a higher risk among male patients [17]. Notably, study population size differences have emerged as a significant factor contributing to variability in reported results.

This nationwide retrospective cohort study used a national database of patients with UC meeting established national diagnostic criteria and included approximately 80,000 participants, indicating sufficient scale. Database quality was high, as specialist-confirmed registrations were subsequently reviewed by local government review committees [18]. Within this study, RR for CRC in UC demonstrated a tendency for females to have higher rates compared to males for both colon and rectal cancer, suggesting that racial and regional differences may also be influencing factors. Conversely, there was no significant difference in family history between the CRC and non-CRC groups.

Although aging and severe inflammation are often associated with UC-related CRC, no significant inflammation type or Mayo score differences were observed at registration. Conversely, CRC was significantly associated with higher frequencies of mucosal friability, erosion, and pseudo-polyps on endoscopy, with histological examinations revealing an exceptionally increased prevalence of dysplasia and abnormal crypt architecture (Table 3).

Regarding CRC risk factors, univariate analysis identified older age at UC onset, presence of pseudo-polyps on endoscopy, and dysplasia as significant factors (Table 4). Multivariate analysis indicated that dysplasia was the only independent risk factor for CRC development. Figure 3 displays an increasing CRC risk trend associated with dysplasia (*p* < 0.001), abnormal crypt architecture (*p* = 0.0001), and pseudo-polyps (*p* = 0.0003). Importantly, dysplasia is recognized as a CRC risk factor owing to its precancerous nature [19]. While pseudo-polyps generally exhibit a weak association with CRC [20], this study indicated that patients with pseudo-polyps demonstrate an increased CRC risk at an earlier stage.

Here, the Mayo score yielded an HR of 0.99 (95%CI: 0.88–1.11, *p* = 0.864) in Cox proportional hazard analysis, indicating no significant difference between the CRC and non-CRC groups, respectively. Conversely, while the Mayo endoscopic score (MES) has been suggested as a new prognostic factor for CRC [21], our study found that the HR for MES demonstrated a significantly higher trend in the CRC group during univariate analysis (HR = 3.14, 95% CI: 1.74–5.65, *p* < 0.001). Nevertheless, in multivariate analysis, the HR adjusted to 0.74 (95% CI: 0.42–1.31, *p* = 0.299), indicating no significant difference. This discrepancy may be attributed to the greater influence of age and dysplasia on CRC risk compared to MES. Overall, these results suggest that MES is useful for screening CRC risk. Conversely, as displayed in Figure 3, the extent of intestinal inflammation has been linked to CRC risk. Therefore, investigating the temporal changes in the Mayo score is necessary. Additionally, abnormal crypt architecture on histological examination was associated with increased CRC risk, observed alongside dysplasia during the remission phase of UC. This finding may serve as a biological marker for inflammation-associated carcinogenesis in the large bowel epithelium in UC [22]. Furthermore, the use of 5-ASA tended to reduce CRC risk (HR = 0.36, 95% CI: 0.18–0.71, *p* = 0.003). If chronic inflammation suppression effectively prevents carcinogenesis, more potent immunosuppressive agents, including corticosteroids, may similarly inhibit CRC development. Nonetheless, the use of corticosteroids, immunosuppressants, and granulocyte/lymphocyte apheresis did not significantly reduce CRC risk (Table 3). Cox proportional hazards analysis showed an HR of 0.83 (95% CI: 0.48–1.43, *p* = 0.515) for corticosteroid use, suggesting that the anti-cancer effects of 5-ASA likely extend beyond its anti-inflammatory properties. 5-ASA is used as a first-line drug for mild to moderate UC patients, and there are already reports indicating that it reduces the risk of colorectal cancer [23]. 5-ASA administration suppresses NF-κB, thereby inhibiting COX-2 transcriptional induction, resulting in decreased PGE2 production. Inflammatory mediator reduction and the resulting decrease in mucosal edema, vasodilation, and cell infiltration may contribute to CRC risk reduction [24]. Clinically, the use of 5-ASA has a clear advantage in promoting mucosal healing in UC, and simultaneously, at the cellular and tissue level, it may suppress the aforementioned cancer-related pathways [25]. Nevertheless, whether 5-ASA selectively suppresses only the excessive activity of EGFR while leaving the necessary repair signals intact remains unclear. Therefore, instead of definitively concluding that 5-ASA is a context-dependent regulator of EGFR, it is more likely that it simultaneously regulates multiple pathways to achieve both repair and antitumor effects [26]. However, the fact that this machine learning study suggests a reduction in CRC risk with 5-ASA use cannot be ignored and provides support for future detailed mechanistic investigations.

Recently, the use of machine learning to validate prognostic factors has become more common [27]. The advantage of predictive models based on machine learning over traditional statistical methods is their ability to incorporate a larger number of explanatory variables while maintaining a certain level of predictive performance. Nonetheless, a challenge remains in demonstrating the correlation coefficients for each explanatory variable in relation to how it influences the outcome, resulting in a “black box” relationship between variables and outcomes [28]. Here, to address this issue, we employed a PWL model that visualizes the relationship between explanatory variables and outcomes [14]. Among prior studies on prognostic factors for CRC associated with UC, none have reported identifying prognostic factors through machine learning applied to medical records. The key factors identified in our machine learning analysis—pseudo-polyps and dysplasia—have already been demonstrated to increase CRC risk in patients with IBD [29]. Meanwhile, 5-ASA has been suggested to potentially reduce CRC risk, which aligns with our findings [30]. The strength of machine learning models lies in their ability to simultaneously model relationships among multiple factors and outcomes, unlike traditional linear models. By combining these factors, we classified patients with UC into clusters based on their CRC risk. This classification distinguished groups that were more prone to developing colon cancer or rectal cancer, or less likely to develop CRC. Previous studies have analyzed CRC risk factors by combining colon and rectal cancers in patients with UC. Nevertheless, by segmenting the population, we identified specific characteristics associated with UC complicated by rectal cancer, such as onset in the 30s, left-sided disease, and a preference for corticosteroid-centered therapy over 5-ASA. Conversely, UC complicated by colon cancer typically presented with onset in the 50s, elevated levels of ESR, C-reactive protein, and white blood cell count, as well as a pattern of total colon involvement. Although CRC risk in UC has been reported to be high in patients under 30 years old, prior studies did not differentiate between colon cancer and rectal cancer, preventing comparison with the results obtained in this study. Conversely, the population in cluster 2, which exhibited the lowest CRC incidence, demonstrated few characteristics that clearly distinguished it from clusters 1 and 3. Notably, the only significant difference was a lower frequency of pseudo-polyps in this cluster.

To understand the clinical epidemiology of rare diseases, tracking patients longitudinally on a national scale using a common registration system is necessary. Consequently, this study conducted an analysis utilizing the intractable disease database provided by the MHLW.

The limitations of this study include its reliance on UC medical information collected annually by the government. Patients participating in this survey may include individuals who only partially meet the diagnostic criteria, as their out-of-pocket medical expenses are subsidized by public funds. Nonetheless, because the MHLW performs automated filtering based on these diagnostic criteria, data reliability is considered to be assured. The national database was limited to the period 2003–2014. Because antitumor necrosis factor alpha antibody treatments for UC became available in Japan in 2010, their effects were not represented in the newly recorded cases from 2003–2006. Current evidence regarding the preventive effects of 5-ASA, biologics, and thiopurines against CRC is inconclusive, warranting further research. Furthermore, the influence of age on reducing CRC risk may be greater than that of these medications [29,30]. In the questionnaires used for the nationwide survey, the presence or absence of cancer was reported in a text-based format, which may have led to incomplete data capture. Additionally, the distinction between colon and rectal cancers was based on the attending physician’s opinion, making the classification ambiguous. Therefore, this study referred to CRC, which encompasses both colon and rectal cancers. Furthermore, in the machine learning analysis of prognostic factors, colon and rectal cancers were not analyzed as separate outcomes. Instead, both were combined as CRC, and UC cases were categorized into three groups: colon cancer only, rectal cancer only, and neither colon nor rectal cancer type. The machine learning model used here was developed exclusively with patients collected in Japan and has not been validated externally across different regions. Consequently, the findings of this study may have regional limitations. Future research should utilize open epidemiological data to facilitate comparisons across different regions.

## 5. Conclusions

Based on national surveillance of patients with UC, we clarified the current status of UC-related CRC. The age of onset for UC-associated CRC peaks in the 30s for both sexes, compared with the general population. The degree of intestinal inflammation has been associated with CRC development, and the detection of dysplasia on biopsy is believed to predict future CRC onset. Additionally, this study demonstrated that pseudo-polyps on endoscopy and dysplasia on biopsied specimens represent risk factors for CRC in patients with UC. Additionally, machine learning applied to overall UC progression revealed that colon cancer is more frequent in patients with pancolitis-type UC and those in their 50s, whereas rectal cancer is more common in patients with proctitis-type UC and those in their 30s. These findings may enhance early detection and prevention of CRC in patients with UC. As a direction for future research, we plan to assess the impact of biologics and molecularly targeted drugs, which were not approved during the study period, on reducing CRC risk using new data from FY2014 onwards. We recognize the limitation that data from FY2014 onwards do not include a system for tracking longitudinal changes over time. As part of our translational research, we also aim to demonstrate potential clinical applications of this model for CRC screening.

## Figures and Tables

**Table 1:** Best hyperparameters for the pointwise linear model in UC clustering.

Hyperparameter	Best Parameter
Number of epochs	250
Number of inner layers	6
Size of layers	170
Batch size	15,085
Level smoothing	0.0069
Learning rate	0.0007
Momentum	0.987
Optimization	Adam
Dropout rate of inner layers	0.04
Dropout rate of input layers	0.23
Regularization coefficient	9.642 × 10^−9^

**Table 2:** UC patient follow-up completion rate for each registration year.

		Number of Patients with Completed Follow-Up									
Registry Year	No. of Registered	1 yr Later	2 yrs Later	3 yrs Later	4 yrs Later	5 yrs Later	6 yrs Later	7 yrs Later	8 yrs Later	9 yrs Later	10 yrs Later
2003	3376	1434 (42.5)	1700 (50.4)	2142 (63.4)	2417 (71.6)	2131 (63.1)	1934 (57.3)	2187 (64.8)	2368 (70.1)	2385 (70.6)	2665 (78.9)
2004	7046	3439 (48.8)	3896 (55.3)	4550 (64.6)	4159 (59.0)	4124 (58.5)	4440 (63.0)	4691 (66.6)	4747 (67.4)	5334 (75.7)	
2005	8482	4651 (54.8)	5465 (64.4)	4819 (56.8)	4266 (50.3)	4837 (57.0)	5307 (62.6)	5299 (62.5)	6340 (74.7)		
2006	6280	3765 (60.0)	3381 (53.8)	2766 (44.0)	3173 (50.5)	3551 (56.5)	3634 (57.9)	4519 (72.0)			
2007	6832	3397 (49.7)	3138 (45.9)	3894 (57.0)	4002 (58.6)	4054 (59.3)	5064 (74.1)				
2008	10,443	4474 (42.8)	5446 (52.1)	5986 (57.3)	6037 (57.8)	7679 (73.5)					
2009	12,534	5325 (42.5)	6296 (50.2)	7048 (56.2)	9397 (75.0)						
2010	13,073	5889 (45.0)	6964 (53.3)	9562 (73.1)							
2011	14,704	6592 (44.8)	9956 (67.7)								
Tracking completion rate (%)		47.9 ±5.8	54.8 ±6.6	59.1 ±7.9	60.4 ±8.9	61.3 ±5.9	63.0 ±6.0	66.5 ±3.5	70.7 ±3.0	73.2 ±2.6	78.9

Yr, year.

**Table 3:** Clinical epidemiology of UC according to CRC status.

	CRC (+)	CRC (−)	
No. Case	141	78,415	*p*-Value
Age	54.5 ± 15.1	41.3 ± 16.5	<0.001
Onset age	50.7 ± 20.1	38.5 ± 16.7	<0.001
Family history (Yes/No)	2/113 (1.8%)	2162/68,789 (3.2%)	0.699
Sex (Male/Female, ratio)	91/50, 1.82	44,570/33,845, 1.32	0.065
BMI	20.3 ± 2.8	22.3 ± 3.5	0.002
Laboratory findings			
RBC (×10^4^/μL)	424 ± 68	447 ± 58	<0.001
Hemoglobin (g/dL)	12.1 ± 2.6	13.1 ± 2.1	<0.001
WBC (/μL)	7338 ± 3246	7058 ± 2944	0.2688
ESR (mm/hr)	28.4 ± 24.8	21.4 ± 25.5	0.041
Total protein (g/dL)	6.9 ± 0.7	7.1 ± 0.7	0.001
Albumin (g/dL)	3.7 ± 0.7	4.1 ± 0.7	<0.001
CRP (mg/dL)	2.3 ± 4.1	1.7 ± 4.3	0.114
Stool culture (positive/negative)	7/88 (7.4%)	4028/51,162 (7.3%)	0.979
Extent of inflammation#			
E1: ulcerative proctitis	7 (5.0%)	4928 (6.3%)	0.140
E2: left-sided colitis	38 (27.0%)	23,133 (29.5%)	
E3: pancolitis	55 (39.0%)	23,743 (30.3%)	
Other	26 (18.4%)	18,002 (23.0%)	
Mayo-score	4.2 ± 2.4	4.4 ± 2.0	0.211
Endoscopic findings			
mucosal friability	110/129 (85.2%)	70,354/75,860 (92.7%)	0.0011
erosion	96/130 (73.8%)	63,912/75,994 (84.1%)	0.0014
pseudo-polyps	30/129 (23.3%)	6480/68,843 (9.4%)	<0.001
Biopsy findings			
crypt abscess	75/117 (64.1%)	48,733/70,174 (69.4%)	0.21
abnormal crypt architecture	67/114 (58.8%)	25,383/68,325 (37.2%)	<0.001
dysplasia	54/116 (46.6%)	4206/68,971 (6.1%)	<0.001
Therapy			
5-ASA	101/138 (73.2%)	72,241/77,037 (93.8%)	<0.001
corticosteroids	33/139 (23.7%)	22,295/74,173 (30.0%)	0.11
immuno-suppressant	0/136 (0%)	1315/71,514 (1.8%)	0.11
G-/L-CAP	2/141 (1.4%)	1002/78,415 (1.3%)	0.88

CRC(+) indicates UC patients who developed CRC. CRC(−) indicates UC patients in whom CRC was not detected. # Montreal classification. 5-ASA, 5-Aminosalicylic acid; Alb, Albumin; BMI, Body mass index; CAP, Cytapheresis; CRP, C-reactive protein; CRC, Colorectal cancer; E1, Ulcerative proctitis; E2, Left-sided colitis; E3, Pancolitis; ESR, Erythrocyte sedimentation rate; G-CAP, Granulocyte apheresis; Hb, Hemoglobin; L-CAP, Leukocyte apheresis; RBC, Red blood cell; WBC, White blood cell.

**Table 4:** Identifying risk factors for colorectal cancer.

	Univariate Analysis			Multivariate Analysis		
Variables	Hazard Ratio	95% CI	*p*-Value	Hazard Ratio	95% CI	*p*-Value
Age	1.05	1.03–1.06	<0.001	1.09	1.03–1.09	<0.001
Age (onset)	1.03	1.01–1.05	<0.001	0.96	0.93–0.99	0.006
Extent of inflammation	1.21	0.79–1.86	0.376			
Mayo score	0.99	0.88–1.11	0.864			
Mayo endoscopic score	3.14	1.74–5.65	<0.001	0.74	0.42–1.31	0.299
Endoscopic findings						
mucosal friability	0.48	0.22–1.01	0.055			
pseudo-polyps	2.92	1.58–5.43	0.001	1.31	0.61–2.84	0.488
Biopsy findings						
abnormal crypt architecture	3.14	1.74–5.65	<0.001	1.82	0.88–3.75	0.106
dysplasia	11.31	6.50–19.69	<0.001	8.53	4.30–16.9	<0.001
Therapy						
5-ASA	0.36	0.18–0.71	0.003	0.52	0.20–1.34	0.182
corticosteroids	0.83	0.48–1.43	0.515			

5-ASA, 5-Aminosalicylic acid; CI, Confidence interval.

**Table 5:** Characteristics of clusters based on machine learning-based prognosis prediction.

	Cluster 1	Cluster 2	Cluster 3	
No. Case Colon Cancer/Rectal Cancer	16,450 88/0	39,040 0/0	23,066 0/53	*p*-Value
Age	53.7 ± 17.0 *	40.4 ± 14.9	33.9 ± 13.2 †	<0.001
Onset age	51.9 ± 17.6 *	37.6 ± 14.9	30.5 ± 12.6 †	<0.001
Sex (Male/Female, ratio)	9058/7392, 1.45 *	21,627/17,413, 1.45 *	13,976/9090, 1.39 †	0.008
Laboratory findings				
RBC (×10^4^/μL)	419 ± 65 †	449 ± 54	463 ± 52 *	<0.001
Hemoglobin (g/dL)	12.4 ± 2.3 †	13.2 ± 2.0	13.6 ± 2.0 *	<0.001
WBC (/μL)	7853 ± 3440 *	7055 ± 2815	6501 ± 2631 †	<0.001
ESR (mm/hr)	36.6 ± 38.3 *	19.3 ± 20.5	14.5 ± 15.7 †	<0.001
Total protein (g/dL)	6.8 ± 0.8 †	7.1 ± 0.6	7.2 ± 0.6 *	<0.001
Albumin (g/dL)	3.7 ± 0.8 †	4.1 ± 0.6	4.2 ± 0.5 *	<0.001
CRP (mg/dL)	2.6 ± 5.2 *	1.5 ± 3.9	1.4 ± 4.1 †	<0.001
Extent of inflammation				
cecum	29.8% *	23.2%	21.6% †	<0.001
ascending colon	31.0% *	23.0% †	24.4%	<0.001
transverse colon	39.2% *	27.5% †	27.6%	<0.001
descending colon	41.8% *	35.7% †	39.7%	<0.001
sigmoid colon	51.5% †	55.1%	66.6% *	<0.001
rectum	12.0% †	15.9%	25.3% *	<0.001
Mayo score	3.9 ± 2.1 †	4.3 ± 2.0	4.8 ± 2.0 *	<0.001
Endoscopic findings				
mucosal friability	88.7% †	93.6%	94.1% *	<0.001
erosion	89.0% *	86.5%	76.4% †	<0.001
pseudo-polyps	9.9% *	7.6% †	9.5%	<0.001
Histopathological findings				
crypt abscess	77.3% *	71.9%	59.0% †	<0.001
abnormal crypt architecture	40.0% *	37.0%	35.0% †	<0.001
dysplasia	4.5% †	5.3%	9.1% *	<0.001
Therapy				
5-ASA	98.0% *	97.0%	85.1% †	<0.001
corticosteroids	21.8% †	27.4%	40.7% *	<0.001
immuno-suppressant	1.5% †	1.7%	2.3% *	<0.001

* Significantly larger (*p* < 0.05) in residual analysis. † Significantly smaller (*p* < 0.05) in residual analysis.5-ASA, 5-Aminosalicylic acid; Alb, Albumin; CRP, C-reactive protein; ESR, Erythrocyte sedimentation rate; RBC, Red blood cell; WBC, White blood cell.

## Data Availability

The MHLW provided anonymized data for analysis (9 March 2021). The original contributions presented in this study are included in the article/Supplementary Material. Further inquiries can be directed to the corresponding author(s).

## Supplementary Materials

The following supporting information can be downloaded at https://www.mdpi.com/article/10.3390/cancers17233752/s1. Table S1: The application form of UC; Table S2: Datasets used in the pointwise linear model; Text S1: Diagnostic criteria for ulcerative colitis.

## Abbreviations

The following abbreviations are used in this manuscript:

FY	Fiscal year
G-CAP	Granulocyte apheresis
L-CAP	Leukocyte apheresis
ADL	Activities of daily living
BMI	Body mass index
RBC	Red blood cell
WBC	White blood cell
ESR	Erythrocyte sedimentation rate
CRP	C-reactive protein
