# Supplementary material for: Analysis of Risk Factors for Colorectal Cancer Associated with Ulcerative Colitis Using Machine Learning: A Retrospective Longitudinal Study Using a National Database in Japan
Source: Cancers (Basel). 2025 Nov 24;17(23):3752. doi: 10.3390/cancers17233752 (PMC12691531; doi:10.3390/cancers17233752)
Supplement: Supplementary file 1 [file cancers-17-03752-s001.zip › cancers-3949005-supplementary.pdf]

cancers

MDPI

Supplementary Materials

Table S1. Diagnostic criteria for ulcerative colitis.

A diagnosis is confirmed when the following conditions are met: (1) is present, one of the items in (2) is satisfied, (3) is present, and the diseases in (4) can be ruled out.

(1) Clinical symptoms

Persistent or recurrent mucous/bloody stool or a history of such stool.

(2) Imaging findings

1) Endoscopy

(a) Diffuse involvement of the mucosa, with a coarse or fine granular appearance, brittleness, and easy bleeding, along with mucous and purulent secretions or

(b) Multiple erosions, ulcers, or pseudo-polyps

2) Intestinal radiography

(a) Diffuse changes on the mucosal surface, such as plasticity or fine granules, or

(b) Multiple erosions, ulcers, or pseudo-polyps, along with disappearance of the haustrum and narrowing or shortening of the intestinal canal.

(3) Biopsy histology

Diffuse inflammatory cell infiltration, mainly in the inner layer of the mucosa, with decreased or absent goblet cells, erosion, crypt abscess, and abnormal glandular arrangement. Even if the examination of (2) and (3) is insufficient or cannot be performed, if gross and histological findings and characteristics of ulcerative colitis are seen during resection surgery or autopsy, the diagnosis is considered to be defined if other diseases (4) can be ruled out.

(4) Diseases that should be ruled out include infection enteritis, such as bacterial dysentery, amoebic dysentery, schistosomiasis japonica, tuberculosis coli, Campylobacter enterocolitis, radiation colitis, ischemic colitis, drug-induced colitis, Crohn's disease, Bechet's intestinal type, and lymph-follicular-proliferative diseases.

Note:

- In some rare cases, patients may not be aware of hemoptysis, so careful consideration is needed.

- If findings are mild and the diagnosis is not specific, it should be treated as a 'suspicious diagnosis' until definite findings are obtained.

Table S1. The application form of UC.

**Item**			**Option**
Application year			YYYY(AD)
Gender			1:Man 2:Woman
Birthday	Year		YYYY(AD)
	Age		
Onset date	Year		YYYY(AD)
	Age		
Date of first visit to a doctor			YYYYMMDD（AD）
Family history			1: Yes 2: No 3: Unknown
Pathological condition	Clinical course	1. First attack	1: Yes
		2. Relapse and remission	1: Yes
		3. Chronic persistent	1: Yes
		If 1 is selected.	1: First 2: Relapse
		4. Acute fulminant	1: Yes
		5. Unknown	1: Yes
	No. of hospitalizations	Total	Current facility + other facilities
		Current facility	
		Other facilities	
	Refractory		1: Yes 0: No
		1. Active for more than 6 months	1: Yes
		2: Relapse at least twice a year	1: Yes
Severity	Date of evaluation		YYYYMMDD（AD）
	Severity		1: mild 2: moderate 3: severe 4: fulminant 5: unknown
Extent of the lesion	Latest affected area	Date of evaluation	YYYYMMDD（AD）
		1. Rectum	1: Yes
		2. Colon	1: Yes
		1. Sigmoid	1: Yes
		2. Descending	1: Yes
		3. Transvers	1: Yes
		4. Ascending	1: Yes
		3. Cecum	1: Yes
		4. Ileum	1: Yes
		5.Unknown	1: Yes
	Intestinal complications		1: Yes 2: No
		If 1 is selected.	Describe the illness.
	Family occurrence of UC		1: Yes 2: No
		If 1 is selected.	Describe the relationship.
	Family occurrence of Crohn disease		1: Yes 2: No
		If 1 is selected.	Describe the relationship.
Internal treatment	Current therapy	1. Steroids	1: Yes 2: No
		2. Intravenous hyperalimentation	1: Yes 2: No
	(Including those associated with postoperative complications.)	3. 5-ASA	1: Yes 2: No
		4. Immunosuppressants	1: Yes 2: No
		5. Other	1: Yes 2: No
		If 1 is selected.	Describe the name of drugs.

	Adverse events			1: Yes 2: No
		If 1 is selected.		Describe symptoms and findings
Surgical treatment	Reason for surgery	1. Bleeding		1: Yes
		2. Megacolon		1: Yes
		3. Cancer		1: Yes
		4. Perforation		1: Yes
		5. Resistant for therapy		1: Yes
		6. Extraintestinal complications		1: Yes
		If 1 is selected.		Describe the detail.
		7. Other		1: Yes
		If 1 is selected.		Describe the detail.
	Date / Surgical method	1st	date of surgery	YYYYMMDD（AD）
			Surgical method	Describe the detail.
		2nd	date of surgery	YYYYMMDD（AD）
			Surgical method	Describe the detail.
	Postoperative complication			1: Yes 2: No
		If 1 is selected.		Describe the detail.
	Macroscopic and histological findings			Describe the detail.
Fecal pathogen microorganism examination	Date			YYYYMMDD（AD）
				1: Yes 2: No
	Name of microorganism	If 1 is selected.		Describe the detail.
Clinical symptoms and findings	Recent findings	Hight (cm)		
		Weight (Kg)		
		1. Main symptoms		
		Date		YYYYMMDD（AD）
		(1) Stool frequency (times/day)		
		(2) Status of stool		
			Bleeding	1: No 2: slightly 3: moderate 4: severe
			Appearance	1: solid 2: muddy 3: watery
		(3) Abdominal symptoms		
			Spontaneous pain	1: Yes 2: No
			Location	Describe the detail.
		(4) Body temperature (℃)		
		(5) Pulse rate (/min)		
		2. Blood test		
		Date		YYYYMMDD（AD）
		Implementation of examination		1: Yes 2: No
		(1) RBC （$\times 10^4$/mm^3）		
		(2) Hemoglobin (g/dl)		
		(3) WBC (/mm^3)		
		(4) ESR (mm)		
		(5)CRP (mg/dl)		
		(6) Total protein (g/dl)		
		(7) Albumin (g/dl)		
		3. Colonoscopically findings		
		Date		YYYYMMDD（AD）
		Implementation of examination		1: Yes 2: No
		(1) Continuous lesion		1: Yes 2: No
		(2) Disappearance of haustra		1: Yes 2: No
		(3) Mucosal fragility		1: Yes 2: No

	(4) Erosion/ulcer	1: Yes 2: No
	(5) Pseudopolyps	1: Yes 2: No
	4. Endoscopically findings	
	Date	YYYYMMDD（AD）
	Implementation of examination	1: Yes 2: No
	(1) Loss of vascular markings	1: Yes 2: No
	(2) Easy bleeding	1: Yes 2: No
	(3) Mucosal fragility	1: Yes 2: No
	(4) Erosion/ulcer	1: Yes 2: No
	(5) Pseudopolyps	1: Yes 2: No
	(6) Continuous lesion	1: Yes 2: No
	5. Biopsy findings (pathological examination)	
	Date	YYYYMMDD（AD）
	Implementation of examination	1: Yes 2: No
	(1) Cell infiltration	1: Yes 2: No
	(2) Erosion	1: Yes 2: No
	(3) Cryptal abscess	1: Yes 2: No
	(4) Reduced no. of goblet cells	1: Yes 2: No
	(5) Mucosal abnormality	1: Yes 2: No
	(6) Dysplasia	1: Yes 2: No
Differential diagnosis	(1) Infectious colitis (bacterial dysentery, amoebic dysentery, schistosomiasis japonica, tuberculosis of the colon, campylobacter enteritis, etc.)	1: Can be distinguished 2: Cannot be distinguished
	(2) Radiational colitis	1: Can be distinguished 2: Cannot be distinguished
	(3) Ischemic colitis	1: Can be distinguished 2: Cannot be distinguished
	(4) Drug-induced colitis	1: Can be distinguished 2: Cannot be distinguished
	(5) Crohn disease	1: Can be distinguished 2: Cannot be distinguished
	(6) Colon Bechet	1: Can be distinguished 2: Cannot be distinguished
	(7) Lymph proliferative disorders	1: Can be distinguished 2: Cannot be distinguished

Table S2. Datasets used in the pointwise linear model.

Feature	Type	Feature	Type	Feature	Type
Gender	B	Stool status	Q	Ed. pseudo-polyps	B
Age	Q	Spontaneous abdominal pain	B	Ed. continuous lesion	B
Age onset	Q	Body temperature	Q	Bp. cell infiltration	B
Daily life	Q	Pulse	Q	Bp. erosion	B
Severity stage	Q	RBC	Q	Bp. cryptal abscess	B
Lesion rectum	Q	Hb	Q	Bp: reduced no of goblet cells	B
Lesion colon	B	WBC	Q	Bp. mucosal abnormalities	B
Lesion sigmoid colon	B	ESR	Q	Bp. dysplasia	B
Lesion descending colon	B	CRP	Q	Tx. corticosteroids	B
Lesion transverse colon	B	TP	Q	Tx. intravenous hyperalimentation	B
Lesion ascending colon	B	Alb	Q	Tx. 5-ASA	B
Lesion cecum	B	Ba. continuous lesion	B	Tx. immunosuppressant	B
Lesion ilium	B	Ba. loss of haustra	B	Tx. CAP	B
Lesion unknown	B	Ba. rough mucosa	B	Tx. anti-TNFα antibodies	B
Distribution (E1,E2,E3,other)	C	Ba. erosion/ulcer	B	Surgery for bleeding	B
Stool bacteria	B	Ba. pseudo-polyps	B	Surgery for cancer	B
Height	Q	Ed. loss of vascular markings	B	Surgery for megacolon	B
Body weight	Q	Ed. easy bleeding	B	Surgery for perforation	B
Stool frequency	Q	Ed. mucosal friability	B	Surgery for resistance	B
Stool bleeding	Q	Ed. erosion	B	Mayo Score	Q

Note: binary variable; B, quantitative variable; Q, category variable; C, barium enema examination; Ba, endoscopic examination; Ed, biopsy examination; Bp, therapy; Tx.
